# An analytical methodology of rock burst with fully mechanized top-coal caving mining in steeply inclined thick coal seam

**DOI:** 10.1038/s41598-024-51207-3

**Published:** 2024-01-05

**Authors:** Pengfei Shan, Zhongming Yan, Xingping Lai, Huicong Xu, Qinxin Hu, Zhongan Guo

**Affiliations:** 1 School of Energy Engineering, Xi’an University of Science and Technology, Xi’an, 710054 Shaanxi China; 2 Key Laboratory of Western Mines and Hazard Prevention of Ministry of Education, Xi’an University of Science and Technology, Xi’an, 710054 Shaanxi China; 3 Department of Civil and Environmental Engineering, University of Strathclyde, Glasgow, G1 1XJ UK; 4 Shaanxi Zhongtai Energy Investment Co., Ltd., Yulin, 719109 Shaanxi China

**Keywords:** Coal, Natural hazards

## Abstract

Rock burst disaster is still one of the most serious dynamic disasters in coal mining, seriously restricting the safety of coal mining. The *b* value is the main parameter for monitoring rock burst, and by analyzing its changing characteristics, it can effectively predict the dangerous period of rock burst. This article proposes a method based on deep learning that can predict rock burst using data generated from microseismic monitoring in underground mining. The method first calculates the *b* value from microseismic monitoring data and constructs a time series dataset, and uses the dynamic time warping algorithm (DTW) to reconstruct the established *b* value time series. A bidirectional short-term and short-term memory network (BiLSTM) loaded with differential evolution algorithm and attention mechanism was used for training, and a prediction model for the dangerous period of rock burst based on differential algorithm optimization was constructed. The study used microseismic monitoring data from the B_1+2_ fully mechanized mining face and B_3+6_ working face in the southern mining area of Wudong Coal Mine for engineering case analysis. The commonly used residual sum of squares, mean square error, root mean square error, and correlation coefficient R^2^ for time series prediction were introduced, which have significant advantages compared to basic LSTM algorithms. This verifies that the prediction method proposed in this article has good prediction results and certain feasibility, and can provide technical support for the prediction and prevention of rock burst in steeply inclined thick coal seams in strong earthquake areas.

## Introduction

Rock burst disasters refer to disasters in which the equilibrium state is destroyed due to mining or other impacts, resulting in the sudden release of energy accumulated inside the coal and rock mass under high stress, which leads to explosive accidents, vibration, and destruction of the coal and rock mass. At present, the rock burst disaster is still one of the most serious dynamic disasters in coal mining. Rock burst disasters may cause casualties. More severe cases can cause surface subsidence, and damage surface structures, farmland, and housing, seriously restricting the protection of resource bases and the sustainability of energy extraction. There have been rock burst disasters in China that destroyed 1000 m of roadway, and the resulting mine earthquake reached 4.3 on the Richter scale^1^. With the development of the country's economy, the supply of energy resources has faced a greater challenge. The mining of shallow coal seams is progressing rapidly, and the depth of coal mining is gradually increasing. Therefore, it is of great significance to effectively predict rock burst events to ensure the safety of coal mine mining and ensure the personal and property safety of underground personnel.

At present, for the prediction of rock burst, experts, scholars and research teams mostly adopt the method of integrating multiple on-site monitoring methods and simulation experiments. DOU et al.^2^ built a cloud platform based on GIS technology, cloud technology, mining geophysics and other technologies, and built an intelligent rock burst risk identification and multi-parameter monitoring and early warning cloud integrated with various monitoring methods such as microseismic, stress, and drilling cuttings. The platform can guide the site to strengthen pressure relief in high-risk areas while giving early warning of impact risks. Some studies obtain the characteristic parameters of acoustic emission, select the monitoring values of microseismic parameters in typical shock mine working faces, analyze the similarity between the characteristics of acoustic emission and microseismic parameters at the laboratory and engineering scales, and establish the characterization of shock acoustic information between the two relationships, so as to effectively predict rock burst^3–6^. To explore the mechanism of far-field low-frequency seismic waves on rock burst, Li et al.^7^ used structural dynamics theory to study the influence of seismic waves on the stability of coal and rock mass from the perspective of resonance and explained the effect of far-field seismic sources on rock burst. reason. Research on the stress change of coal and rock mass, ground stress change, mining rock mass rupture law, and microseismic monitoring of dynamic and static stress through various technologies has also made progress in providing simple and effective methods for rock burst risk analysis and evaluation^8–11^. To solve the predicament of poor generalization ability and insufficient mining of massive data features in the current rock burst prediction method based on physical indicators, some expert teams combined deep learning technology to initially try to establish a rock burst model driven by the fusion of physical indicators and data features. Time series forecasting methods^12,13^. Zhu^14^ analyzed the stress change and energy release law of the advancing and fractured areas of the working face and revealed the rock burst mechanism under the coupling effect of the square working face and the regional tectonic stress, providing a basis for the prevention and control of square rock burst in the working face under the condition of regional tectonic stress. A theoretical basis is provided. Multiple microseismic data has been used by experts and scholars to identify the precursory information of coal and rock burst, and based on this, the rock burst events can be effectively predicted and warned^15^. With the integration of artificial intelligence methods and traditional mining problems, the continuous improvement of the theoretical system of intelligent rock mechanics has provided scientific and effective prediction methods and means for rock burst^16^, and the deep learning algorithm has been introduced into rock burst prediction. Perfect prediction algorithms for rock burst prediction have gradually become the focus of current research^6,12,13,17^.

At present, the methods for predicting rock burst are relatively complicated, the requirements for personnel are high in practical applications, and the prediction accuracy of the prediction model using deep learning is low. At present, the bidirectional long-term short-term memory network (BiLSTM) model with a good prediction effect has a gating unit and a strong nonlinear mapping ability, which can solve the problem of long-term dependence of data features in prediction research and the problem of bidirectional data feature extraction. The parameters required for model establishment can also be calculated from the microseismic monitoring data. This paper introduces the optimized BiLSTM neural network model based on the microseismic monitoring data, which can automatically complete the *b* value calculation through a numerical fitting software, and quickly predict the dangerous period of rock burst, providing an effective forecasting reference indicator.

## Engineering background overview and data sources

### General situation of engineering background of Wudong coal mine

Wudong Coal Mine, affiliated with China National Energy Group Xinjiang Energy Co., Ltd., was established on January 5, 2013. Wudong Minefield is located in the southeast section of Zhunnan Coalfield, about 34 km northeast of Urumqi City and 13 km north of Midong New District. The administrative division is under the jurisdiction of Midong New District of Urumqi City. The area of the wellfield is about 20.28km^2^, the geological resources in the wellfield are 1.28 billion tons, and the designed recoverable reserves are 661 million tons. In the southern mining area of Wudong Coal Mine, the coal-bearing strata in the minefield using inclined shafts, multi-levels, and zonal development methods are the Middle Jurassic Xishanyao Formation, which is distributed in a northeast-southwest direction. The southern area includes two extremely thick coal seams, B_1+2_ and B_3-6_. The average thickness of the B_1+2_ coal seam is 37.45 m. The B_3-6_ coal seam is located the north of the B_1+2_ coal seam, with a distance of 53–110 m and an average thickness of 48.87 m. B_1+2_ and B_3-6_ coal seams are steeply inclined coal seams with an average dip angle of 87°. The Wudong coal mine has been mined to a level of 425. With the increase of mining depth, the number of rock burst events increases, and the probability of high-energy rock burst events increases, which restricts the sustainability of coal resource extraction in coal mines.

**Figure 1:**
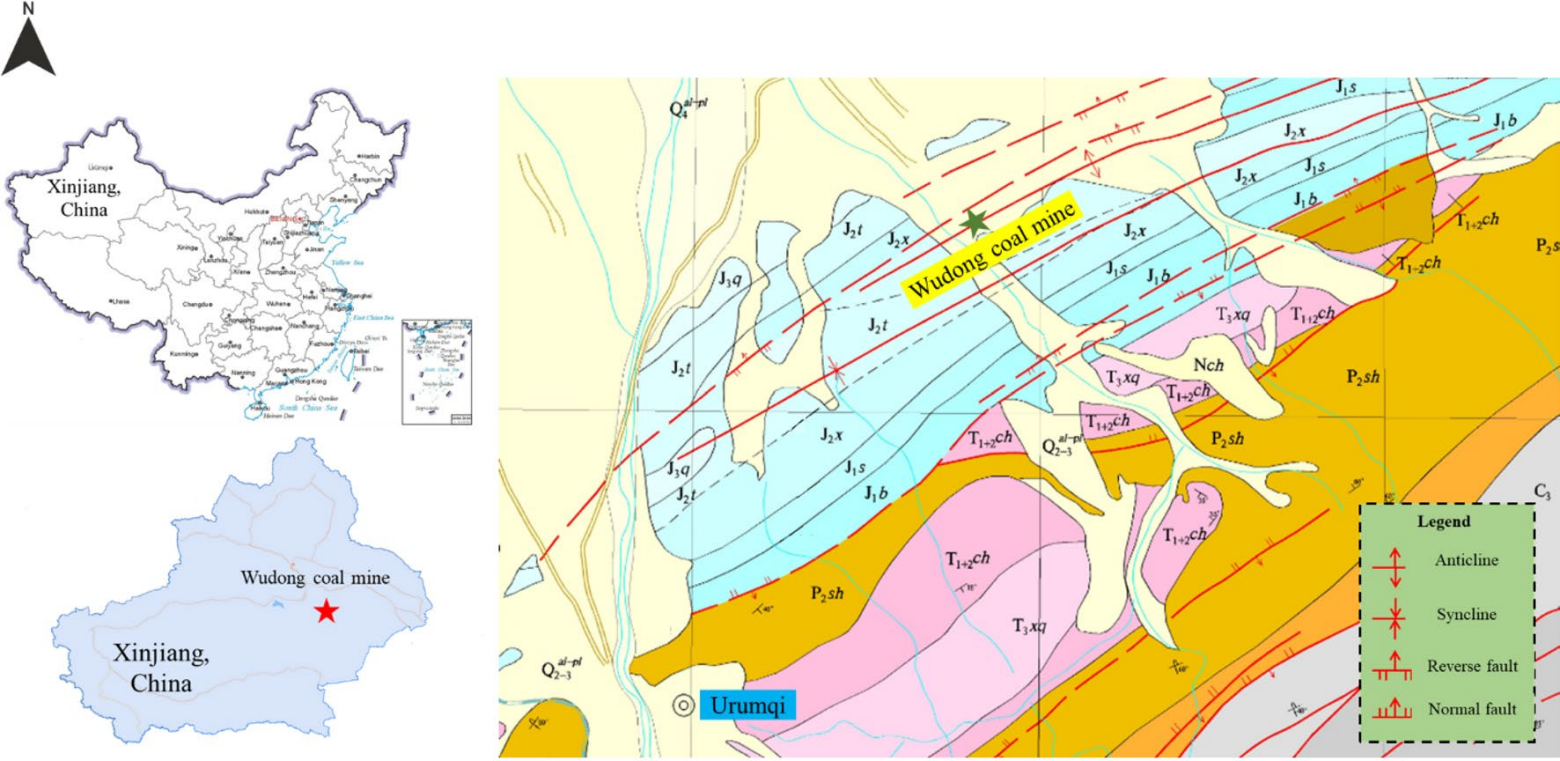
Geographical location map of Wudong Coal Mine.

**Figure 2:**
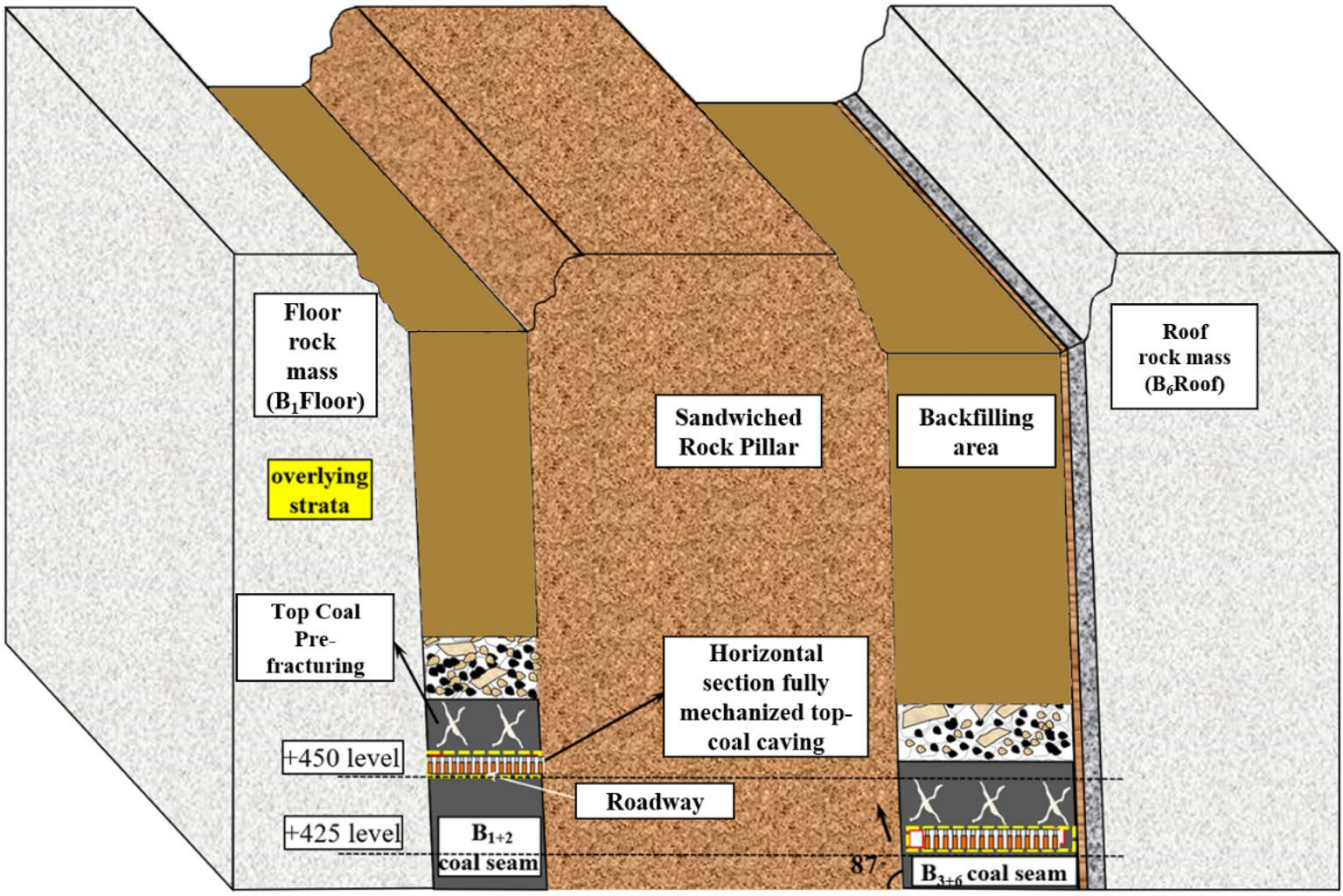
Sketch map of coal seam mining in southern mining area.

The geographic location map of Wudong Coal Mine and the coal seam mining situation in the south mining area are shown in Figs. 1 and 2.

### Data sources

**Figure 3:**
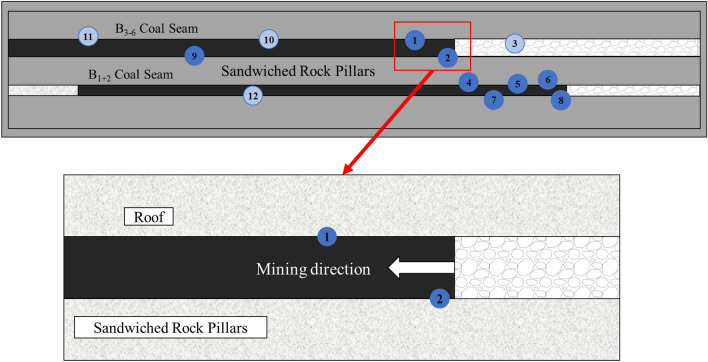
Microseismic sensor layout diagram.

In this paper, the microseismic monitoring data in the south mining area of Wudong Coal Mine are used for research. Wudong Coal Mine adopts the ARAMIS M/E microseismic monitoring system developed by the Polish Institute of Innovative Technology. The system can automatically collect and filter microseismic signals, record the occurrence time of rock burst, calculate the released energy and three-dimensional coordinates, and evaluate the possibility of rock burst and the impact risk area. The sampling frequency of the system sensor is 500 Hz, and the sensitivity is 110 Vs/m10%. The system can monitor low-frequency high-energy microseismic events with energy greater than 100 J and a frequency range of 0–150 Hz, with a positioning accuracy of ± 20 m (X, Y), ± 50 m (Z). A total of 12 sensors have been installed in the south mining area of Wudong Coal Mine, surrounding the research face. Each sensor covers a monitoring range of about 2 km. During the advancing process of the + 450 horizontal fully mechanized caving face of the B_3 + 6_ coal seam, three of the sensors (labeled 1, 2 and 9) move with the working face, and other sensors are arranged near other working faces and roadways. The sensor layout diagram is shown in Fig. 3:

Based on the ARAMIS M/E microseismic monitoring system deployed on site, a total of 89,762 pieces of microseismic monitoring data from Wudong Coal Mine from 2013 to 2019 were collected. The data source samples are shown in Table 1. This paper screens the source data based on the occurrence of rock burst events in the southern mining area of Wudong Coal Mine.

**Table 1:** Source data sample.

Date	X	Y	Z	Energy	Working face/Mining area/coal seam
2016-10-24 05:50:04	1906	4420	483	2.40E + 02	+ 425B6 Driving face East wing B3-6 W
2016-10-24 06:42:43	1113	4135	487	1.90E + 04	+ 475B1 + 2 Working face East wing B1 + 2 W
2016-10-24 07:22:05	1141	4448	487	3.00E + 02	+ 425B6 Driving face East wing B3-6 W
2016-10-24 08:45:41	2673	5195	458	2.00E + 02	+ 450B3 + 6 Working face East wing B3-6 W
2016-10-24 12:46:48	1192	4431	456	2.40E + 04	+ 425B6 Driving face East wing B3-6 W
2016-10-24 13:33:47	1182	4430	459	6.10E + 03	+ 425B6 Driving face East wing B3-6 W
2016-10-24 14:33:29	1086	4237	448	5.00E + 02	+ 475B1 + 2 Working face East wing B1 + 2 W
2016-10-24 14:54:11	2651	5167	458	7.90E + 02	+ 450B3 + 6 Working face East wing B3-6 W
2016-10-24 14:57:05	2651	5178	460	2.90E + 04	+ 450B3 + 6 Working face East wing B3-6 W
2016-10-24 16:41:21	1142	4437	445	1.10E + 03	+ 425B6 Driving face East wing B3-6 W
2016-10-24 17:15:00	1195	4265	448	1.80E + 03	+ 475B1 + 2 Working face East wing B1 + 2 W
2016-10-24 18:33:53	1182	4448	454	8.50E + 03	+ 425B6 Driving face East wing B3-6 W
2016-10-24 19:26:06	1093	4221	448	5.50E + 03	+ 475B1 + 2 Working face East wing B1 + 2 W
2016-10-24 19:54:22	1137	4435	479	6.90E + 03	+ 425B6 Driving face East wing B3-6 W
2016-10-24 20:19:40	1198	4400	450	1.60E + 02	+ 425B6 Driving face East wing B3-6 W

## Microseismic monitoring data processing

### Calculation method of physical index

The* b* value was first derived from the Gutenberg–Richter law in seismology, also known as the G–R relationship. Its formula is:

1 $$\lg N = a - bM$$

Among them, *N* represents the number of earthquake events whose magnitude is greater than *M*, and *a* and *b* are constants^18^.

The numerical change of *b* value is significantly related to the large-energy rock burst event. It is a commonly used physical index in the research of rock burst prediction. It can be used as a judgment index to reflect and measure the internal rupture event of rock mass, and is widely used in the prediction method of rock burst. At present, the calculation of *b* value mainly uses the maximum likelihood estimation method and the least squares method. The maximum likelihood estimation method of *b* value was proposed by Utsu^19^ in 1965. Its formula is:

2 $$b = \frac{N\lg e}{{\sum\limits_{i = 1}^{N} {(M_{i} - M_{0} )} }}$$

where *N* is the total number of events; *M*_*i*_ is the magnitude of the ith microseismic event; *M*_*0*_ is the minimum magnitude of data sampling.

The calculation method of the linear least square method for *b* value is as follows:

3 $$b = \frac{{\sum\limits_{i = 1}^{m} {M_{i} } \cdot \sum\limits_{i = 1}^{m} {\lg N_{i} } - m\sum\limits_{i = 1}^{m} {M_{i} } \cdot \lg N_{i} }}{{\left( {\sum\limits_{i = 1}^{m} {M_{i} } } \right)^{2} - m\sum\limits_{i = 1}^{m} {M_{i}^{2} } }}$$

where *N* is the total number of events; *M*_*i*_ is the magnitude of the *i*-th microseismic event; *N*_*i*_ is the number of microseismic events with the *i*th level of energy.

Considering that the *b* value is predicted as time series data in this paper, the linear least square method is used to calculate the *b* value in actual calculation.

### The establishment of *b* value time series

A time series is a set of random variables sorted by time, which is usually the result of observing a certain underlying process at a given sampling rate in an equally spaced period. Time series data essentially reflects the trend of one or some random variables changing over time, and the core of time series forecasting methods is to mine this law from the data and use it to estimate future data.

The *b* value data is obtained by statistically calculating the microseismic monitoring data with the linear least squares method through the fitting software. In the actual calculation, the appropriate number of energy bins will be selected for statistical calculation with the step size of days, and the *b* value time series will be constructed in units of days, which will be used as the data set studied in this paper.

### *b* value time series feature reconstruction based on dynamic time warping

The calculation of the *b* value is to some extent influenced by the number and location of microseismic events monitored by monitoring equipment, and there are some avoidable errors in actual calculations. Therefore, we divided and screened the data collected during the entire mining process in the southern mining area of Wudong Coal Mine. Based on the monitored microseismic high-energy events and recorded historical rock burst events, the data from the first thirty days and the last ten days of the event from a time series and are numbered. In actual screening, the time window may be adjusted based on the intensity of the target microseismic event and the actual mining progress.

The filtered multi-segment *b* value time series will be introduced into the dynamic time warping algorithm for similarity matching. Assuming S_1_ and S_2_ are two time series with lengths of n and m, respectively. Compare two-time series using a matrix, and use the elements in the matrix to represent the Euclidean distance between two points on the time series. The DTW algorithm will search for a minimum path from (1,1) to (n, m), which satisfies certain boundary conditions, continuity, and monotonicity. The algorithm specifies a minimum distance is:

4 $$\gamma (i,j) = d(S_{i}^{1} ,S_{j}^{2} ) + \min \left\{ {\left. {\gamma (i - 1,j),\gamma (i,j - 1),\gamma (i - 1,j - 1)} \right\}} \right.$$

Due to the fact that the dynamic time warping algorithm can match multiple indices on other sequences with the indices on a single sequence when calculating the shortest distance, it effectively avoids the error of singular points in monitoring data caused by measures such as stopping production or reducing production in the actual monitoring of microseismic monitoring systems. The maximum limit matches the variation characteristics of the *b* value time series before the occurrence of rock burst events and high-energy microseismic events. Through the DTW dynamic regularization algorithm, *b* value time series with obvious features and high similarity can be selected as a training set for the subsequent construction of deep learning models, improving the accuracy of prediction results.

### Ethics approval

The authors declare that this article does not involve human or animal experiments and does not require ethics approval.

## CNN-BiLSTM-attention predictive model building

### Bidirectional long-term short-term memory network model

Long short-term memory neural network(LSTM) is a time recurrent neural network suitable for processing and predicting important events with relatively long intervals and delays in time series.

LSTM is composed of input $X_{t}$ at time t, cell state $C_{t}$, temporary cell state $\tilde{C}_{t}$, hidden state $h_{t}$, forget gate $f_{t}$, memory gate $i_{t}$, and output gate ${\text{o}}_{t}$. The calculation process of LSTM can be summarized as follows. By forgetting the information in the cell state and memorizing new information, the useful information for subsequent calculations can be transmitted, while the useless information is discarded, and the hidden layer state $h_{t}$ is output at each time step. Among them, forgetting, memory and output are controlled by the forgetting gate $f_{t}$, memory gate $i_{t}$, and output gate $o_{t}$ calculated by the hidden layer state $h_{t - 1}$ at the last moment and the current input.

**Figure 4:**
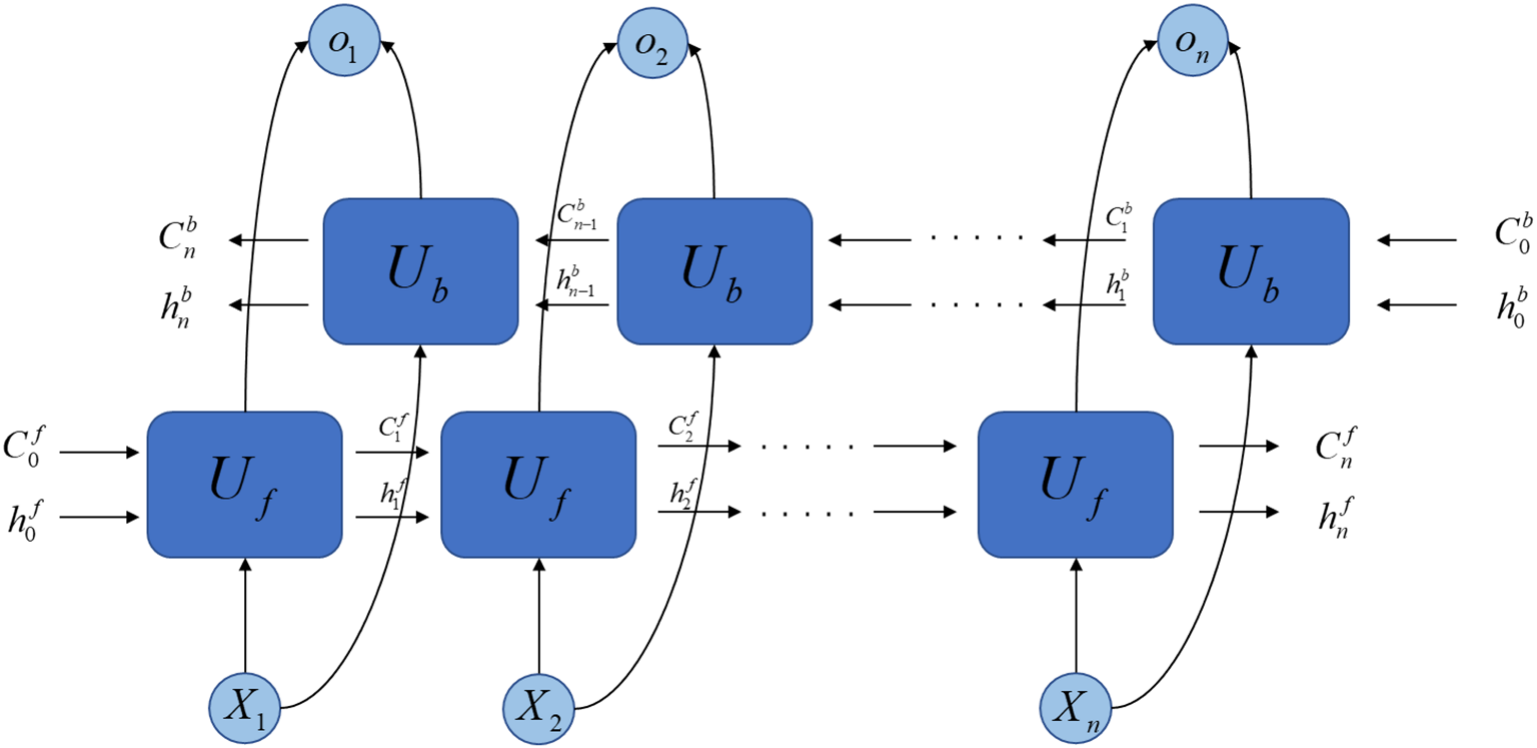
BiLSTM data flow diagram.

In practical applications, it is usually composed of multiple unit modules, and a chain structure is formed between multiple units. Similar to the calculation process of LSTM, the two-way long short-term memory network adds reverse operations on its basis. Added a cell state data flow for passing back information. This can be understood as reversing the input sequence and recalculating the output in the same way as LSTM. The final result is a simple stack of the result of the forward LSTM and the result of the reverse LSTM. It can realize the recursion and feedback of time series data, integrate the data before and after the time series, and improve the data utilization rate. It is also more sensitive to the time characteristics of the time series, and has better prediction accuracy than the LSTM model in time series prediction. The BiLSTM data flow diagram is shown in Fig. 4:

In the data flow diagram, $U_{f}$ represents the forward LSTM layer, and $U_{b}$ represents the reverse LSTM layer. In the layer, each time a unit is forwardly calculated, the output value of the forward hidden layer at the current moment is calculated. In the same way, each unit in the $U_{f}$ layer is reversely calculated once, and the output value of the backward hidden layer at the current moment is calculated. At time *t*, the output of the BiLSTM network is the sum of $h_{t}^{f}$ and $h_{t}^{b}$ values, which constitutes the overall hidden layer state of the BiLSTM network $h_{t}$. $C_{n}^{b}$ and $C_{n}^{f}$ represents the forward or backward cell state to the next unit.

Its formula is:

5 $$h_{t}^{f} = LSTM\left( {X_{t} ,h_{t - 1}^{f} } \right)$$

6 $$h_{t}^{b} = LSTM\left( {X_{t} ,h_{t - 1}^{b} } \right)$$

LSTM represents the calculation of the aforementioned LSTM unit, and the parameters represent the current input and the hidden state of the current forward or reverse unit. Based on this, the overall hidden layer state of the BiLSTM network at time t is obtained:

7 $$h_{t} = W^{f} h_{t}^{f} + W^{b} h_{t}^{b}$$

where $W^{f}$ and $W^{b}$ are the weights of the output value of the forward hidden layer and the output value of the backward hidden layer at time t in the calculation of the overall shadow hidden layer state of the network.

### Optimization of prediction model by attention mechanism

When using a deep learning model for time series forecasting, the model usually needs to receive and process a large amount of data for training and forecasting. In the case of limited computing power, computing resources need to be allocated to more important tasks. At the same time, the problem of information overload needs to be addressed. The attention mechanism is introduced as an allocation scheme to focus information on the input data set of the model, identify key information and allocate computing resources to enhance effective features. It can also reduce the attention to other data and suppress invalid features, filter invalid data, and solve the problem of data overload. At the same time, it can also improve the efficiency of model training and the accuracy of model prediction.

Consider the input information vector *h* as an information storage, and now given a query vector *q* to find and select some information in *h*, then you need to know the index position of the selected information. A "soft" selection mechanism is adopted to extract some features from all the information, and the most relevant information is extracted more.

Define an attention variable $z \in \left[ {1,N} \right]$ to indicate the index position of the selected information, that is, $z = i$ indicates that the *i*th input information is selected, and then calculate the probability of selecting the *i*-th input information given *q* and *h*. The calculation formula as follows:

8 $$\alpha_{i} = p\left( {z = i\left| {h,q} \right.} \right)$$

Use the softmax function to normalize the results:

9 $$\alpha_{i} = {\text{softmax}}\left( {s\left( {h_{i} ,q} \right)} \right){ = }\frac{{\exp \left( {s\left( {h_{i} ,q} \right)} \right)}}{{\sum {_{j = 1}^{N} \exp \left( {s\left( {h_{j} ,q} \right)} \right)} }}$$

The probability vector formed by $\alpha_{i}$ becomes the attention distribution. It indicates how relevant the i-th information in the input information vector *h* is to the query *q* when a query *q* is given. For the attention scoring function $s\left( {h,q} \right)$, there are several specific forms shown in Table 2.

**Table 2:** Attention scoring function model.

Functional model	Formula
Additive model	$s\left( {h,q} \right) = v^{T} \tanh \left( {Wh + Uq} \right)$
Dot product model	$s\left( {h,q} \right) = hq$
Scaling dot product model	$s\left( {h,q} \right) = \frac{{h^{t} q}}{\sqrt d }$
Bilinear model	$s\left( {h,q} \right) = h^{T} Wq$

Where *W*, *U*, and *v* are learnable network parameters, and *d* is the dimension of the input information. In specific use, select the appropriate attention scoring function according to the characteristics of the data set.

After the attention distribution $\alpha_{i}$ is calculated, the soft information selection mechanism is used to give the result of the query, that is, the input information is summarized by weighted average to obtain the Attention value, and the final feature vector can be calculated according to the following formula:

10 $$F = att\left( {h,q} \right) = \sum\limits_{i = 1}^{N} {\alpha_{i} h_{i} }$$

### Time-series prediction model of rock burst *b* value based on differential algorithm optimization

Differential evolution algorithm is a heuristic random search algorithm based on group differences, proposed by Storn and Price in 1995, aimed at solving Chebyshev polynomials. The differential evolution algorithm has a simple principle, few controlled parameters, and strong robustness, and is widely used in the optimization of deep learning algorithms. The differential evolution algorithm designs genetic operators by simulating hybridization, mutation, and replication in genetics. The algorithm mainly includes three steps mutation, crossover, and selection. The mutation vector is generated by the difference vector of the parent generation, and crosses with the individual vector of the parent generation to generate a new individual vector, which is directly selected with the individual of the parent generation.

**Figure 5:**
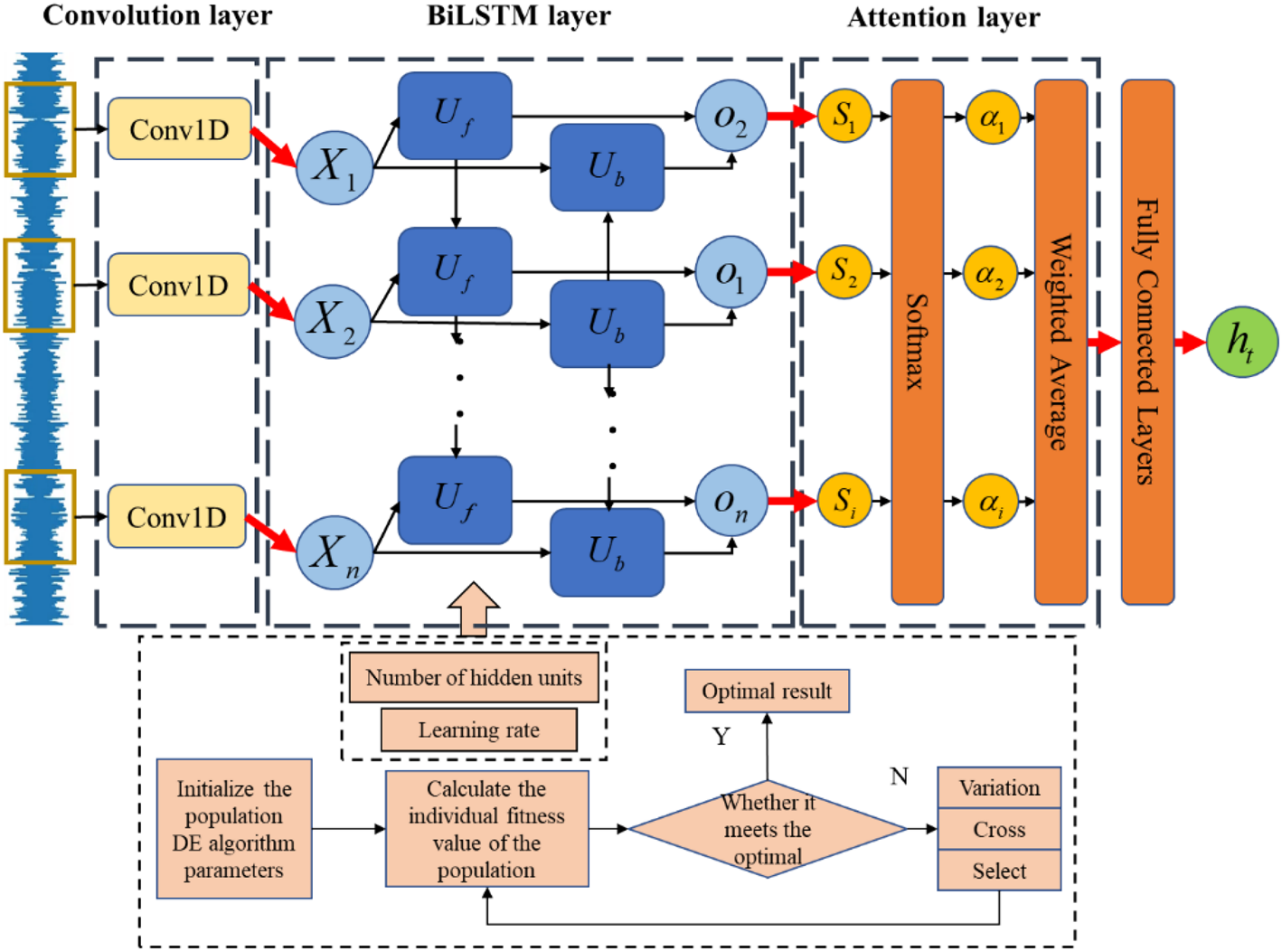
Multi-variable temporal prediction model of rock burst based on difference algorithm.

Since the CNN-BiLSTM-Attention algorithm does not integrate a hyperparameter optimization algorithm, manual adjustment of hyperparameters is prone to overfitting, which affects the quality of the trained model and the ability of the model to infer correct results on new inputs. The automatic hyperparameter optimization algorithm can select appropriate hyperparameters to ensure the best performance of the neural network and avoid the impact of manual adjustment of hyperparameters on algorithm performance. Therefore, this paper adds a differential algorithm to the constructed CNN-BiLSTM-Attention model to optimize the number of hidden units and learning rate of BiLSTM to ensure better model training results while reducing running time and memory budget. The structure diagram of the model constructed in this paper is shown in Fig. 5.

The CNN algorithm will first perform feature extraction on the time series dataset. Firstly, take time series data as input and extract features through one or more convolutional layers. Each convolutional layer can have multiple convolution kernels, each of which can extract different features.

## Example application for microseismic data

### Data processing

Data were screened based on historical rock burst events in the south mining area of Wudong Coal Mine. The microseismic monitoring data of 30 days before and 10 days after the occurrence of previous rock burst events are used as the data set of this paper. A total of 30,672 pieces of microseismic monitoring data were selected from March 2015 to April 2017 at the 475-level B_3-6_ working face, 450-level B_3-6_ working face and 450-level B_1+2_ fully mechanized mining face. The basic information of the five historical rock burst events is shown in Table 3

**Table 3:** Rock burst event in the southern mining area of Wudong Coal Mine.

Occurrence time	Event energy/J	Source location
2015.03	4.9 × 10^8^	+ 487Level Clamp the rock column
2015.07	9.0 × 10^7^	+ 470Level Clamp the rock column
2016.11	9.5 × 10^6^	+ 460Level Clamp the rock column
2017.02	2.1 × 10^8^	+ 460Level B_6_ Roof
2017.04	2.2 × 10^6^	+ 431Level Clamp the rock column

Preliminary calculations were carried out after screening the data, and the *b* value was calculated. The geological conditions and mining technology of each mine are different, and the microseismic monitoring data of different mines and even different mining faces of the same mine are also different. Therefore, when evaluating the *b* value, it is necessary to select the energy range ∆E, and select microseismic events within the appropriate magnitude range to evaluate the *b* value. Aiming at the problem of selecting the energy range, according to the microseismic monitoring data in the south mining area of Wudong Coal Mine, with 0.2 as the energy classification, statistical analysis of the difference between the distribution characteristics of the monitoring data and the G–R linear relationship, a reasonable range of microseismic energy values is obtained.

In order to select the appropriate energy range to reflect the actual situation of the relationship between the energy and the number of events in the mine, according to the representative rock burst events in July 2015 and February 2017, the microseismic monitoring data of the adjacent months and the month when the event occurred were selected Make statistics and obtain the trend of microseismic monitoring data to determine the selected energy range.

**Figure 6:**
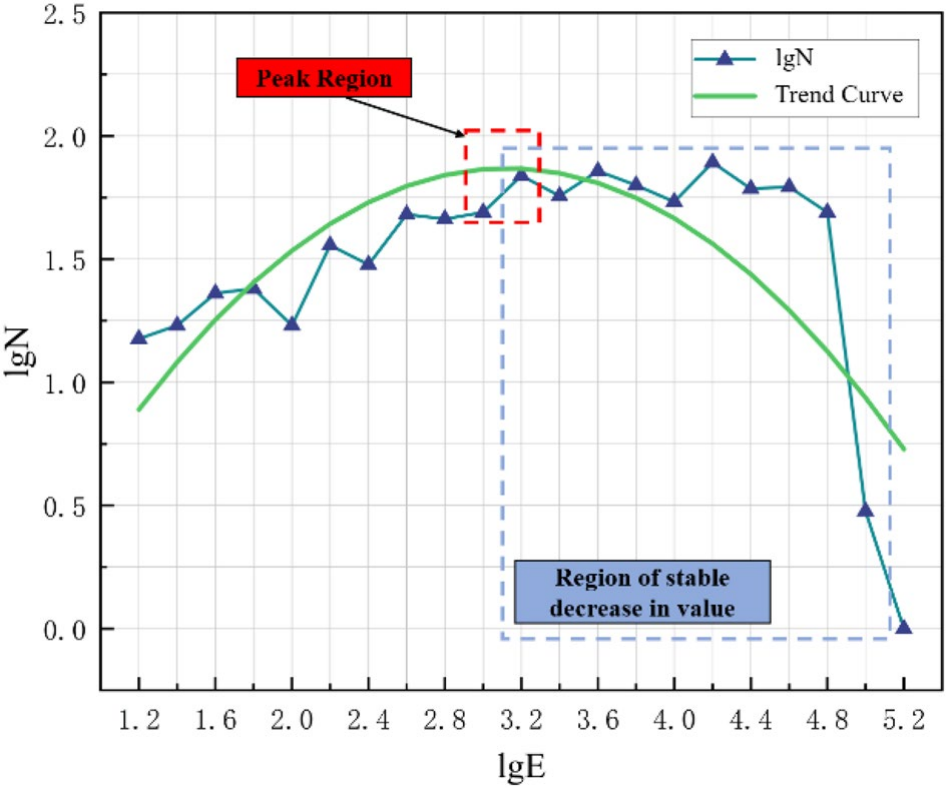
Trend chart of microseismic monitoring data in July 2015.

**Figure 7:**
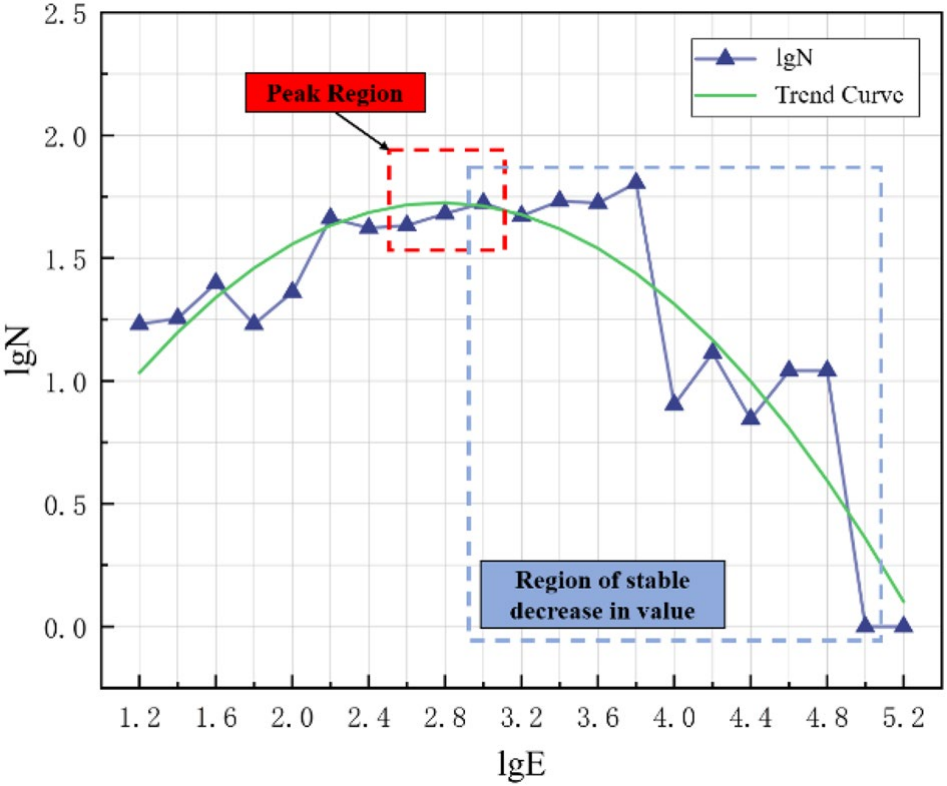
Trend chart of microseismic monitoring data in February 2017.

As shown in Figs. 6 and 7, the analysis of the fitting curve shows that when lgN is at the peak, the value of lgE is between 2.6 and 3.0. At the same time, when the value of lgE is between 2.8 and 4.8, lgN maintains a steady downward trend. This trend has certain unary function characteristics. To sum up, the selected energy range is 2.6 ≤ lgE ≤ 4.8, which is used as the value interval for studying the relationship between energy and event number.

### Model training and evaluation

After screening, a total of 7 *b* value time series were obtained. The time spans of the seven time series are shown in Table 4:

**Table 4:** Multiple time series datasets.

Series label	Time span
1	February 17, 2015 to March 29, 2015
2	June 3, 2015 to July 13, 2015
3	September 15th, 2015 to October 8th, 2015
4	April 7th, 2016 to May 19th, 2016
5	January 21, 2017 to March 2, 2017
6	March 21, 2017 to April 30, 2017
7	August 23, 2017 to November 1, 2017

Introduce the DTW dynamic time warping algorithm to perform similarity testing on multiple time series mentioned above. Calculate the minimum path weighted length between two arbitrary time series separately as the residual between the time series, and evaluate the similarity based on the time series residual.

**Figure 8:**
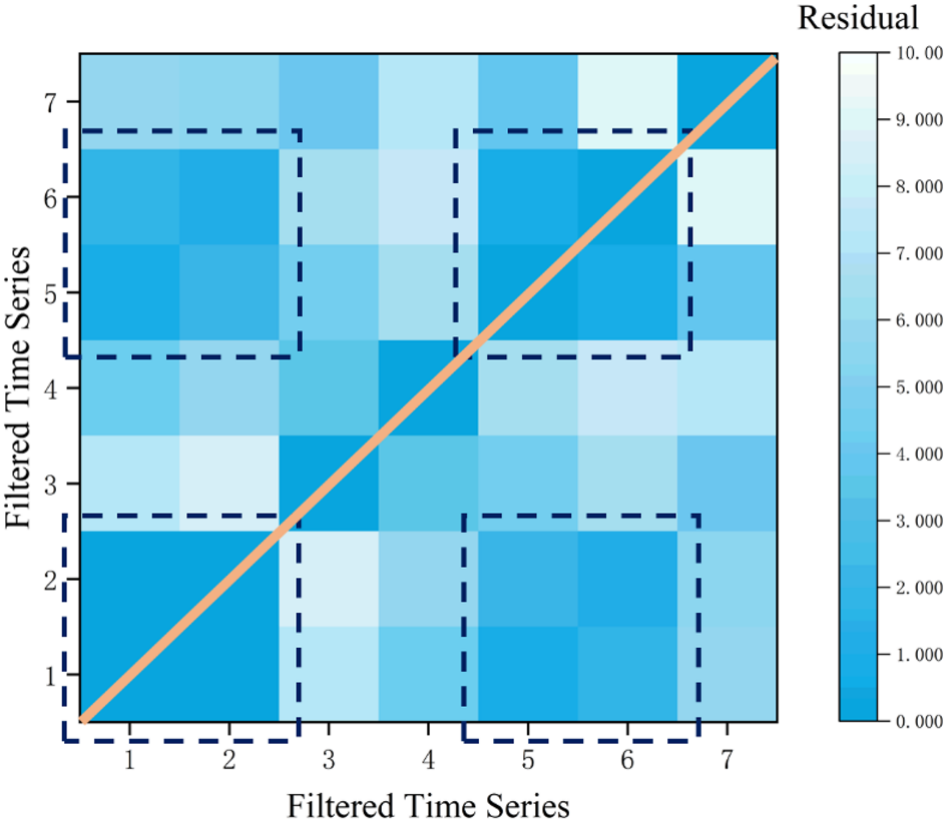
Time series similarity.

The time series similarity matrix is shown in Fig. 8.

Through inspection, it can be found that S_1_, S_2_, S_5_, and S_6_ have a certain degree of similarity and clustering, with relatively similar and obvious features.

Based on the above results, S_1_ and S_2_ are used as the training set, S_5_ is the validation set, and S_6_ is the test set. The hyperparameters of the BiLSTM algorithm were optimized by the difference algorithm, and the optimization results of the model were analyzed to obtain the optimal model for time series prediction of rock burst *b* value in Wudong Coal Mine. Determine the parameter values of the difference algorithm as shown in the following Table 5.

**Table 5:** Parameter setting of difference algorithm.

Parameter	Value
Population size	25
Iteration rounds	40
Shrinkage factor	0.5
Crossover rate	0.5
Vector value range	− 100,100

The differential algorithm is used to find the optimal solution for the hyperparameters of the rock burst prediction model, and the prediction model is trained with the training set to obtain the hyperparameter values of the deep learning model with the best fitness value. According to the model training results, the number of BiLSTM layers is set to 2 layers, and the number of neurons in each layer is set to 189 and 128. The learning rate is initially set to 0.008, and the algorithm dynamically decays to the minimum value of 0.0001 when it has converged.

### Performance analysis

To test the optimization effect of automatic parameter tuning in differential algorithms, we selected another common hyperparameter optimization algorithm—genetic algorithm—as a comparison. The minimum value of loss rate convergence in the iterative loop is shown in Table 6. Compared to not adding differential algorithm optimization and using genetic algorithm optimization, using differential algorithm optimization significantly reduces the loss value.

**Table 6:** The lowest value when the loss rate converges.

Algorithm	Value	Comparison value
Differential algorithm	0.025	–
Default	0.13	80.8%↑
Genetic algorithm	0.055	54.5%↑

**Figure 9:**
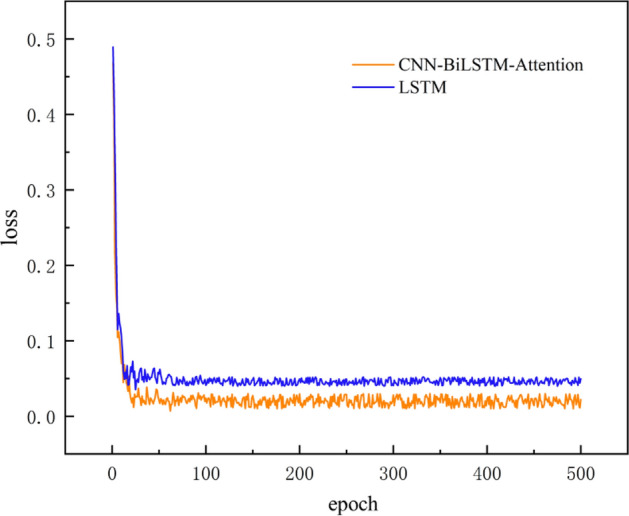
Prediction model loss curve.

To determine the model optimization effect and verify whether the prediction effect of the model can meet expectations, this paper selects the traditional LSTM algorithm and compares it with the deep learning model used in this paper. Both models use the same dataset as the training set. In order to intuitively reflect the difference between the prediction models, MSE is selected as the loss function, and the number of iterations is set to 500. Visually compare the loss curves of the prediction models. The loss curve is shown in Fig. 9.

It can be seen intuitively from Fig. 9 that the two prediction models have reached convergence within fifty times and tended to a dynamic stable state. The average loss value of the CNN-BiLSTM-Attention algorithm used in this paper is 1.86 × 10^–3^, and the average loss value of the original LSTM algorithm is 4.66 × 10^–3^.

In this paper, when evaluating the performance of the model, two common performance indicators, root mean square error (RMSE) and correlation coefficient (also known as the coefficient of determination or coefficient of determination R^2^), are considered. These two metrics are often used and representative when evaluating time series forecasting models. In the formula, $I$ represents the actual value of the microseismic monitoring physical index, $\widehat{I}$ represents the predicted value of the microseismic monitoring physical index, $i=\left(\mathrm{1,2},\cdot \cdot \cdot N\right)$.

The RMSE mathematical expression is:

11 $$RMSE = \sqrt {\frac{1}{N}\sum\limits_{i = 1}^{N} {(\hat{I}_{i} - I_{i} )^{2} } }$$

The R^2^ mathematical expression is:

12 $$R^{2} = 1 - \frac{{\sum\limits_{i = 1}^{N} {(\hat{I}_{i} - I)^{2} } }}{{\sum\limits_{i = 1}^{N} {(I_{i} - \overline{I})^{2} } }}$$

**Figure 10:**
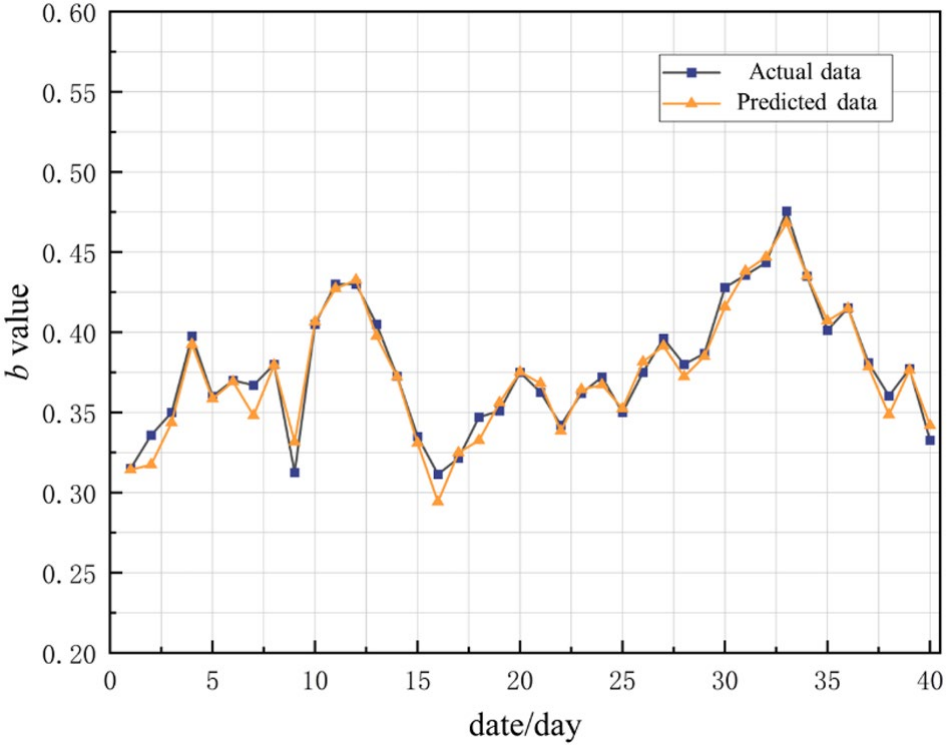
Prediction rendering.

Taking the microseismic monitoring data from January 21, 2017 to March 2, 2017 as the verification set, the trained model is used to predict the *b* value during this period, and the actual value is compared and analyzed. The predicted value and the actual value are shown in Fig. 10.

**Figure 11:**
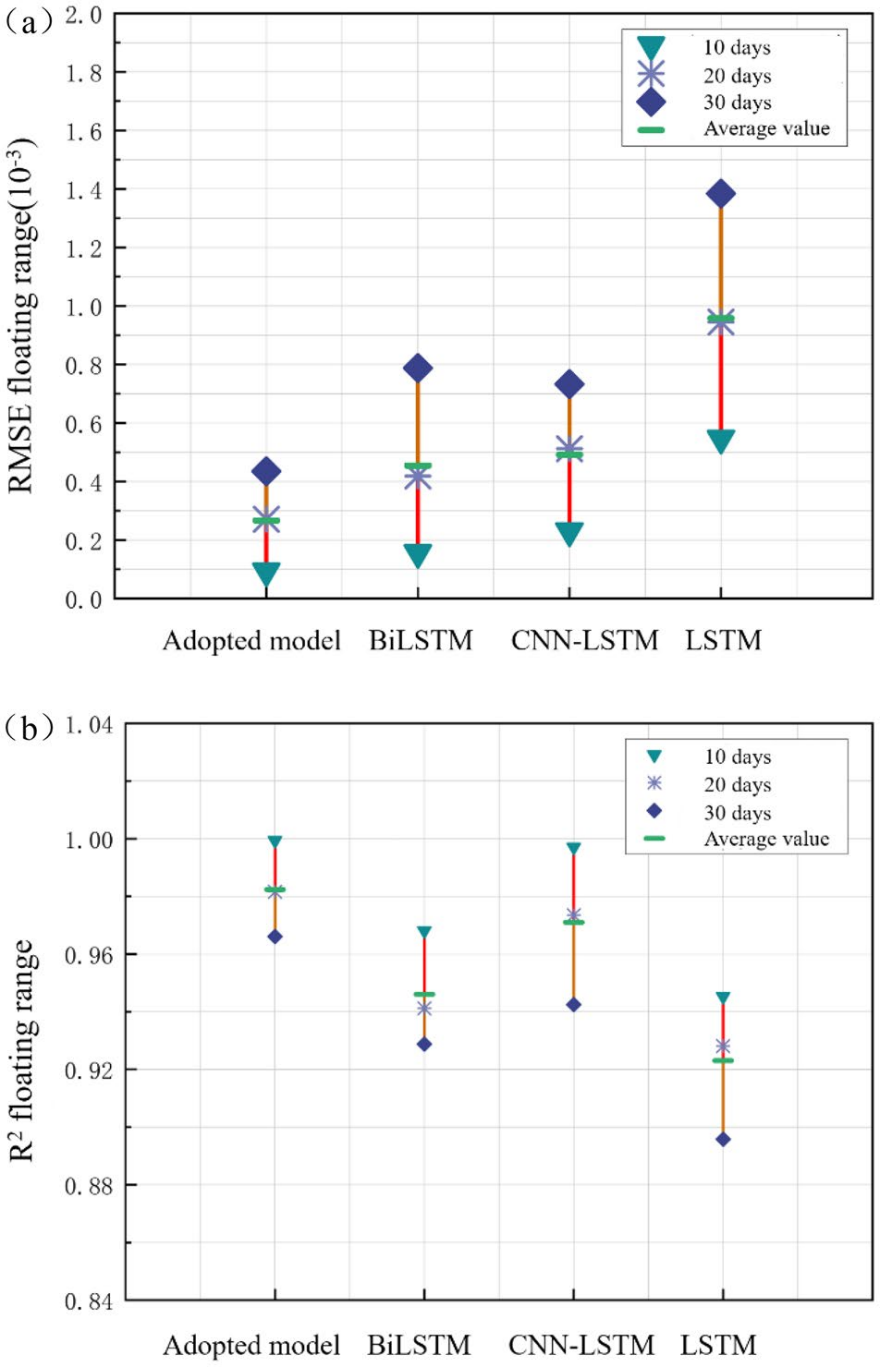
(**a**) Comparison of prediction effect of RMSE. (**b**) Comparison of prediction effect of R^2^.

Calculate the parameter value of the performance evaluation index. Select the same data and use the same calculation method to analyze the prediction effect of the classic LSTM model, BiLSTM model, and CNN-LSTM model, and calculate RMSE and R^2^ with the span of 10 days, 20 days, and 30 days as the prediction time. Comparing the prediction performance indicators of the four models, the results are shown in Fig. 11.

Figure 11 clearly shows that the RMSE of the models used in this article is lower than other models, and R^2^ is higher than other models. The prediction effect is better when the prediction duration is 10 days, but the prediction effect gradually decreases with the increase of time span. In actual prediction, the prediction duration is adjusted according to specific needs.

### Rock burst prediction based on trained model

According to the above model training and evaluation, and model performance analysis, it can be seen that the multivariate time-series prediction model of rock burst based on differential algorithm optimization established in this paper has good predictive performance and can accurately predict large-energy rock burst events. Predict the *b* value from March 21, 2017 to April 30, 2017, and use the aforementioned prediction method to predict the dangerous period that may occur in the future period of rock burst events. The *b* value is used for prediction. When the model predicts that the *b* value drops below the average value of this period, it is regarded as an early warning indicator that triggers a rock burst event. When predicting time series, we found that the algorithm did not experience severe overfitting by adjusting the retrospective window to compare the prediction performance, confirming that the algorithm used in this article obtained this change feature. And finally confirm whether the early warning event should be taken seriously, and further determine the key safety precaution period of the rock burst event.

**Figure 12:**
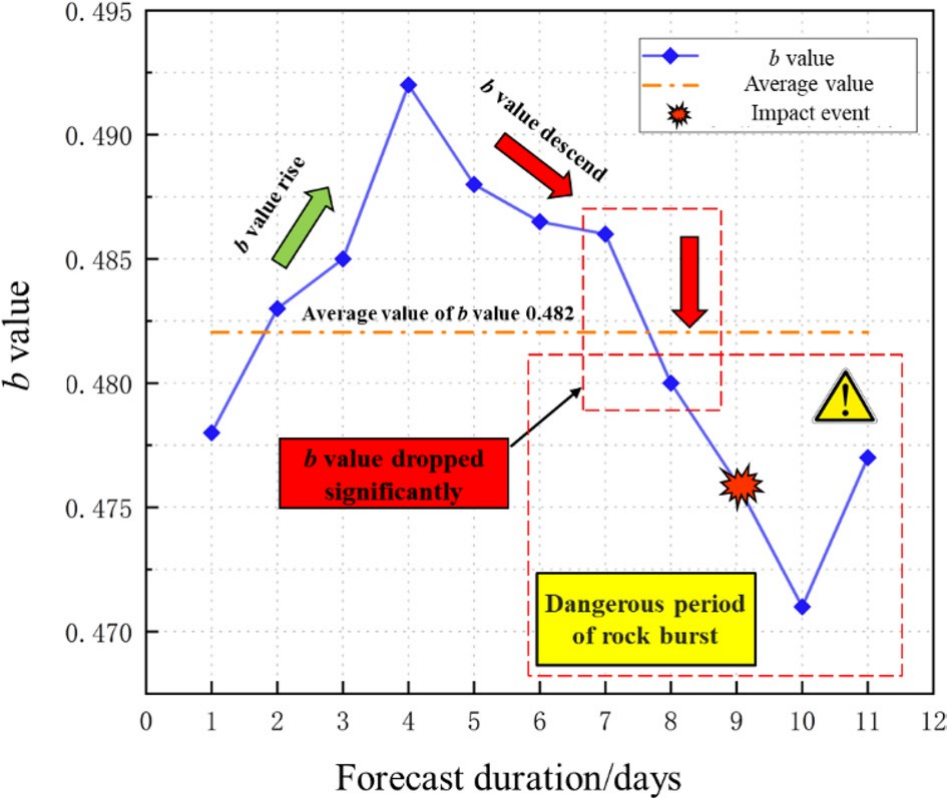
Project example prediction effect diagram.

As can be seen from Fig. 12, the predicted value shows that the *b* value first rises steadily and then shows a downward trend within the forecast period, and then suddenly drops when it falls, and the value drops below the average value within the period to trigger an early warning. Therefore, the time period when *b* value drops sharply and *b* value rises back to the average value is evaluated as the rock burst dangerous period. April 10, 2017 is the 9th day predicted by the model in the figure. A large-energy rock burst event occurred on that day, which was within the dangerous period of rock burst predicted by the model.

## Conclusion

1. By determining a reasonable energy level division based on the collected microseismic monitoring data, the *b* value data was automatically calculated using the linear least squares method through fitting software, and a *b* value time series dataset was constructed. The established time series was reconstructed using the dynamic time warping algorithm (DTW), which maximized the extraction of *b* value changes in rock burst and high-energy microseismic events, providing reliable data support for model training and prediction.
2. A multi-variable time-series prediction model of rock burst based on differential algorithm optimization is established, and the differential algorithm is introduced to optimize the hyperparameters of the model, and a time-series prediction model of rock burst based on differential algorithm optimization is established. The forecasting effect of the model was verified by comparing the four performance evaluation indicators commonly used in time series forecasting, RMSE and R^2^.
3. The predictive model is used to predict and analyze future rock burst events, which verifies the feasibility and reliability of the model established in this paper for actual rock burst prediction. After verification, this method can predict rock burst events in coal mining under steep inclined environments, and can provide data support for safe, sustainable, and efficient mining of coal mines. The geological conditions and mining techniques of each mine are different, and the microseismic monitoring equipment used in each mine is also different. Therefore, in the future, model training will be expanded based on the data collected from different mines and different working faces, and try to obtain the universal characteristics of the change trend of *b* value in rock burst events.

## Data Availability

The data presented in this study are available on request from the corresponding author.
